# Whole-genome sequencing of *Aminobacterium* sp. strain MB27-C1, isolated from the wastewater treatment plant of a steel mill

**DOI:** 10.1128/mra.00073-24

**Published:** 2024-03-11

**Authors:** Junlin Huang, Chih-Hung Wu, Chao-Jen Shih, Yen-Chi Wu, Shu-Jung Lai, Yi-Ting You, Sheng-Chung Chen

**Affiliations:** 1 College of Chemical Engineering, Fuzhou University, Fuzhou, Fujian, China; 2 School of Resources and Chemical Engineering, Sanming University, Sanming, Fujian, China; 3 Fujian Provincial Key Laboratory of Resources and Environmental Monitoring and Sustainable Management and Utilization, Sanming University, Sanming, Fujian, China; 4 Bioresource Collection and Research Center, Food Industry Research and Development Institute, Hsinchu, Taiwan; 5 Graduate Institute of Biomedical Sciences, China Medical University, Taichung, Taiwan; 6 Research Center for Cancer Biology, China Medical University, Taichung, Taiwan; 7 Department of Life Sciences, National Chung Hsing University, Taichung, Taiwan; Rochester Institute of Technology, Rochester, New York, USA

**Keywords:** sewage sludge, *Aminobacterium*, steel mill, rolling-tube method, anaerobes

## Abstract

Here, we report the complete genome sequence of *Aminobacterium* sp. strain MB27-C1, which was isolated from sewage sludge collected at the wastewater treatment plant of Sanming Steel Co. Ltd. in Fujian, China. The resulting genome of strain MB27-C1 is a single contig of 2,427,830 bp with 41.58% GC content.

## ANNOUNCEMENT

**Fig 1:**
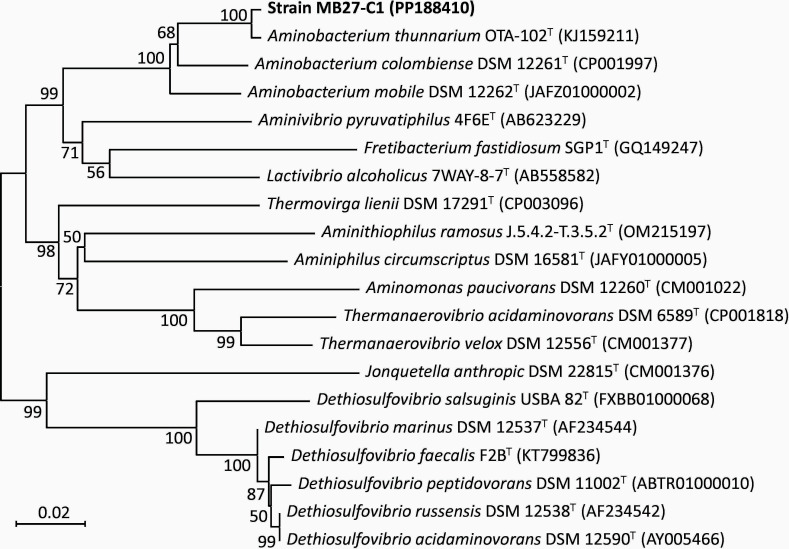
Neighbor-joining tree based on 16S rRNA gene sequences of strain MB27-C1 and related taxa. The phylogenetic tree was constructed using MEGA11 software (6). Bar, 0.02 substitutions per nucleotide position. Bootstrap values were expressed as percentages of 1,000 replications.

Strain MB27-C1 was isolated from sewage sludge collected from the wastewater treatment plant of Sanming Steel Co. Ltd., located in Fujian, China, on 25 June 2021. Approximately 1 mL of sludge was directly inoculated into liquid anaerobic modified DSM924 media without acetate and formate. After incubation at room temperature (~25°C) for 2 weeks, the isolation, purification as well as 16S rRNA amplification and sequencing were conducted on the enrichment culture according to the methods described in our previous study (1). Based on the analysis using Nucleotide BLAST (2), one of the isolates, designated as strain MB27-C1, showed the highest similarities to three validly characterized strains: *Aminobacterium thunnarium* OTA 102^T^ (NR_178662.1, 98.47%) (3), *Aminobacterium colombiense* ALA-1^T^ (NR_074624.1, 95.92%) (4), and *Aminobacterium mobile* ILE-3^T^ (NR_024925.1, 91.25%) (5). In addition, phylogenetic analysis of 16S rRNA gene sequences performed by MEGA11 (6) for strain MB27-C1 and related taxa indicates that strain MB27-C1 belongs to the genus *Aminobacterium* (Fig. 1). The genome of strain MB27-C1 was sequenced for species delineation and comparative genomic analysis.

Strain MB27-C1 was cultured in modified DSM 924 medium at 35°C for 2 days. The genomic DNA was extracted from a cell pellet collected from a 1-L culture using the NucleoBond HMW DNA kit (Macherey-Nagel, Germany) following the manufacturer’s instructions. The genome was sequenced at the Sangon Biotech (Shanghai) Co., Ltd. using the DNBSEQ-T7 platform (MGI Tech Co., Ltd.) and the MinION sequencer (Oxford Nanopore Technology).

For the DNBSEQ-T7 platform, sheared genomic DNA fragments of approximately 300 bp were employed to construct a 150-bp paired-end DNA library. The library preparation was performed using Hieff NGS MaxUp II DNA Library Prep Kit for Illumina from Yeasen Biotechnology (Shanghai) Co., Ltd. Subsequently, the constructed library underwent sequencing using MGISEQ-2000RS High-throughput Sequencing Set (PE150 format), generating a total of 11,227,458 reads, which were then trimmed using Trimmomatic v0.36 (7). In the case of the MinION sequencer, the sheared DNA fragments underwent a series of processing steps, including end repair, 3′ adenylation (NEBNext End Repair/dA-Tailing Module), and ligation to adaptors pre-loaded with motor proteins (NEBNext Quick Ligation Module). The resulting product was then purified using Agencourt AMPure XP Beads (Beckman, A63881). Subsequently, fragments larger than 1 kb were subjected to single-molecule nanopore DNA sequencing using the Multiplex Ligation Sequencing Kit (SQK-MLK111.96-XL) on a MinION Flow Cell (R9.4.1). Basecalling was performed using Guppy version 6.5.6 with the default model, yielding a total of 115,526 reads and *N*_50_ of 6,328 bp. Trimming of the reads was carried out using Porechop v0.2.4 (8), and filtering was executed with NanoFilt v2.8.0 (9). DNBSEQ-T7 and MinION reads were hybrid *de novo* assembled using Canu v2.2 (10), producing a circularized contig of 2,427,830 bp with 41.58% GC content. Gene predictions and annotations were performed using NCBI Prokaryotic Genome Annotation Pipeline v6.5 (11). The antibiotics and secondary metabolite analysis shell (antiSMASH) was used to mine biosynthetic gene clusters in the genome (12). Default parameters were used for all bioinformatics analyses. Table 1 provides the basic characteristics of this strain and genome.

**TABLE 1:** Basic characteristics of *Aminobacterium* sp. strain MB27-C1 based on MIGS (Minimum Information for Genome Sequence) recommendation and general information on its genome

Item	Description
MIGS data	
NCBI BioProject	PRJNA1006156
NCBI BioSample	SAMN37010248
GenBank accession number	CP133089
Geographic location	Sewage sludge of wastewater treatment plant of Sanming Steel Co. Ltd., Sanming, Fujian, China
Latitude and longitude	26.2649 N, 117.6245 E
Collection date	25 June 2021
Sequencing platforms	DNBSEQ-T7 and Oxford Nanopore MinION
Assembly method	Canu v. 2.2
Coverage	1,018.0×
Finishing strategy	Complete
General features of strain	
Classification	Domain: *Bacteria* Phylum: *Synergistota* Class: *Synergistia* Order: *Synergistales* Family: *Synergistaceae* Genus: *Aminobacterium*
Gram stain	Positive
Cell shape	Rod-shaped
Relationship to oxygen	Anaerobic
Optimal growth temperature	40°C
Optimal growth pH range	7
Optimal growth NaCl concentration	1.0%
Genomic features	
Size (bp)	2,427,830 bp
GC content (%)	41.58
CDSs^*b*^	2,272
Number of tRNAs	48
Number of rRNAs	3, 3, 3 (5S, 16S, 23S)
antiSMASH result	One RiPP-like gene cluster^*a*^

^*a*^ RiPP, ribosomally synthesized and post-translationally modified peptide.

^*b*^ CDSs, Coding DNA Sequences.

## Data Availability

The genome sequence of strain MB27-C1 has been deposited in GenBank under accession number CP133089. The version of the genome described in this paper is the first version. The BioProject and BioSample accession numbers are PRJNA1006156 and SAMN37010248. DNBSEQ-T7 and MinION raw reads were deposited in the Sequence Read Archive (SRA) under accession numbers SRR27217864 and SRR27217863, respectively. The 16S rRNA gene sequence of strain MB27-C1 has been deposited in GenBank under the accession number PP188410.
